# Susceptibility for Some Infectious Diseases in Patients With Diabetes: The Key Role of Glycemia

**DOI:** 10.3389/fpubh.2021.559595

**Published:** 2021-02-16

**Authors:** Jesús Chávez-Reyes, Carlos E. Escárcega-González, Erika Chavira-Suárez, Angel León-Buitimea, Priscila Vázquez-León, José R. Morones-Ramírez, Carlos M. Villalón, Andrés Quintanar-Stephano, Bruno A. Marichal-Cancino

**Affiliations:** ^1^Departamento de Fisiología y Farmacología, Centro de Ciencias Básicas, Universidad Autónoma de Aguascalientes, Aguascalientes, Mexico; ^2^Facultad de Ciencias Químicas, Universidad Autónoma de Nuevo León, Nuevo León, Mexico; ^3^Centro de Investigación en Biotecnología y Nanotecnología, Facultad de Ciencias Químicas, Universidad Autónoma de Nuevo León, Nuevo León, Mexico; ^4^Unidad de Vinculación Científica de la Facultad de Medicina, Universidad Nacional Autónoma de México en el Instituto Nacional de Medicina Genómica, Mexico City, Mexico; ^5^Departamento de Farmacobiología, Cinvestav-Coapa, Mexico City, Mexico

**Keywords:** infections, diabetes, immune system, hyperglycemia, COVID-19

## Abstract

Uncontrolled diabetes results in several metabolic alterations including hyperglycemia. Indeed, several preclinical and clinical studies have suggested that this condition may induce susceptibility and the development of more aggressive infectious diseases, especially those caused by some bacteria (including *Chlamydophila pneumoniae, Haemophilus influenzae*, and *Streptococcus pneumoniae*, among others) and viruses [such as coronavirus 2 (CoV2), Influenza A virus, Hepatitis B, etc.]. Although the precise mechanisms that link glycemia to the exacerbated infections remain elusive, hyperglycemia is known to induce a wide array of changes in the immune system activity, including alterations in: (i) the microenvironment of immune cells (e.g., *p*H, blood viscosity and other biochemical parameters); (ii) the supply of energy to infectious bacteria; (iii) the inflammatory response; and (iv) oxidative stress as a result of bacterial proliferative metabolism. Consistent with this evidence, some bacterial infections are typical (and/or have a worse prognosis) in patients with hypercaloric diets and a stressful lifestyle (conditions that promote hyperglycemic episodes). On this basis, the present review is particularly focused on: (i) the role of diabetes in the development of some bacterial and viral infections by analyzing preclinical and clinical findings; (ii) discussing the possible mechanisms by which hyperglycemia may increase the susceptibility for developing infections; and (iii) further understanding the impact of hyperglycemia on the immune system.

## Introduction

Diabetes mellitus is a chronic and complex illness characterized by several metabolic alterations including dyslipidemia and hyperglycemia, among others (1). According to the American Diabetes Association (A.D.A.), diabetes mellitus (DM) can be classified into the following categories: (i) type 1 diabetes mellitus (T1DM), characterized by the loss of pancreatic β-cells induced by an autoimmune response; (ii) type 2 diabetes mellitus (T2DM), identified by the gradual loss of insulin secretion and/or the development of insulin resistance; (iii) gestational DM, developed in some pregnant women; and (iv) other types of DM that are due to miscellaneous causes (2, 3). Interestingly, patients with uncontrolled DM (regardless of type) have alterations in healing latency and susceptibility for developing some emerging infectious (mainly bacterial) diseases. In addition, compared to non-diabetic normoglycemic patients, DM patients are at higher risk for developing the current severe acute respiratory syndrome coronavirus 2 (SARS-COV2) caused by the coronavirus 2 (CoV2) that has shocked the world economy and created a global health pandemic emergency named COVID-19 (4–7). Moreover, the restoration of normoglycemia seems to be related to a better prognosis for bacterial infections (5); whereas in COVID-19 diabetic patients, no obvious conclusions have been reached about the impact of normoglycemic treatments on the development and outcome of this particular disease (8).

People with metabolic impairments (i.e., fasting hyperglycemia, postprandial hyperglycemia and DM) show greater ranges of glucose levels (2). Indeed, fasting hyperglycemia (when food has not been taken for at least 8 h) is a metabolic disorder characterized by levels of plasma glucose above 110 mg/dL, a condition commonly observed in diabetic patients (9). Fasting hyperglycemia (from now on simply referred to as hyperglycemia) has been involved in deleterious effects such as tissue damage associated with oxidative stress and immunological impairments (10), which increase the susceptibility to acquire bacterial infections and COVID-19 (8, 11–17) (see below).

**Figure 1:**
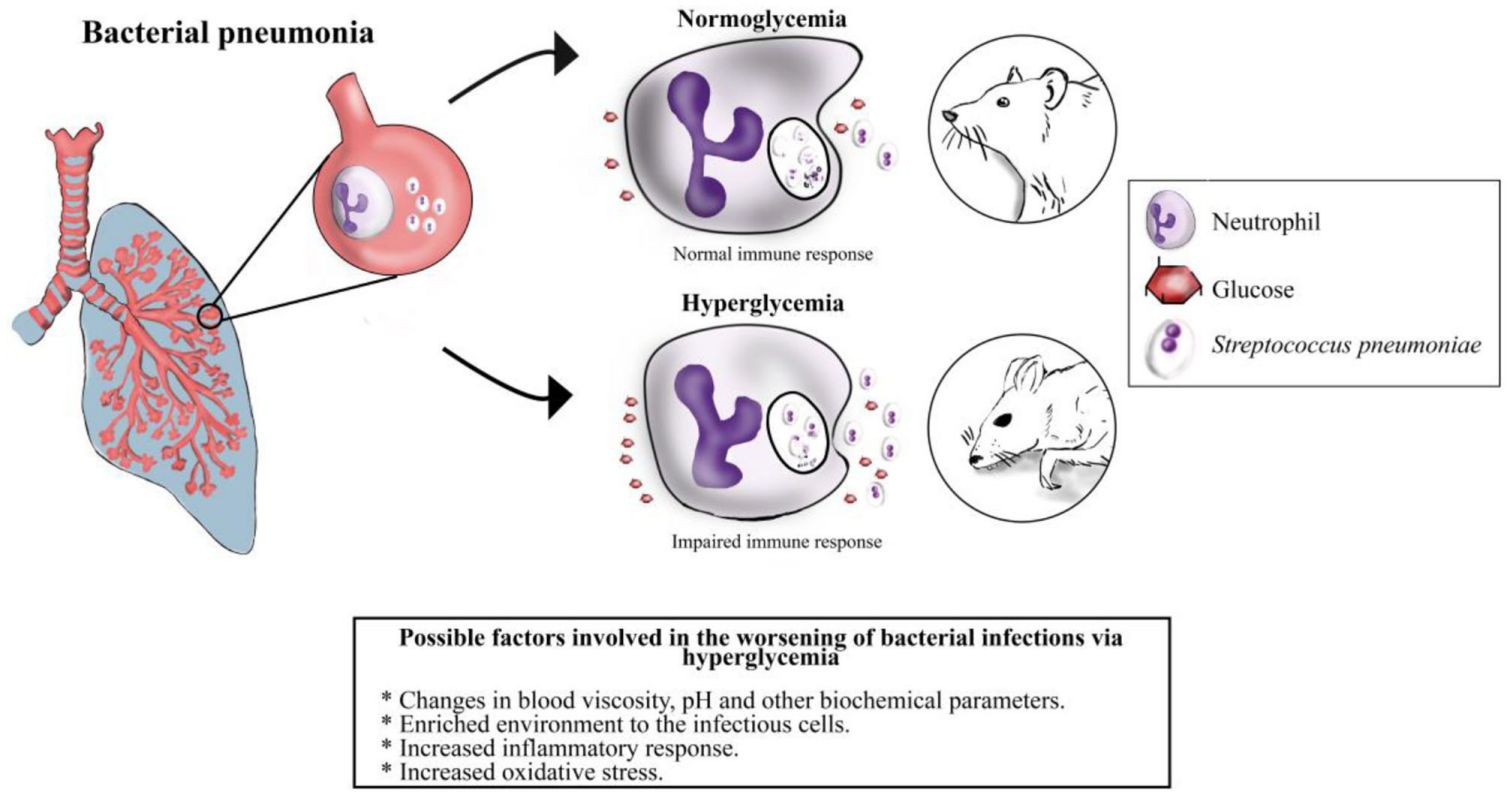
Illustration of a hypothetical outcome in an experimental model of bacterial pneumonia in normal conditions vs. during hyperglycemia. Under normoglycemia conditions the immune response handles successfully bacterial infections. Nevertheless, hyperglycemia impairs the immune response by inducing several glucose-related factors, including those mentioned above. These scenarios could determine the outcome during bacterial infections.

Remarkably, the effects of hypoglycemia induced by anorexia on the clinical outcome of infected patients have been discussed with no consensus (18). Moreover, Wang et al. (19) have suggested that: (i) the pathogenic nature (i.e., bacterial, viral, etc.) and infection profile may be key factors for the prognosis and clinical outcome in a preclinical model of bacterial infection; (ii) glucose plays a key role in the outcome of infected animals; and (iii) survival of animals under bacterial sepsis (with *Listeria monocytogenes*) was dramatically decreased when they were gavage-fed. In contrast, if these animals received glucose (i.p.), all animals died (19). As anorexia is an important stereotypic behavior of the *sickness response*, it could be an adaptive strategy for combating some infectious illnesses. In this sense, it has also been reported that bacteria from other groups (e.g., *Salmonella*) may induce inhibition of anorexia via *Salmonella* leucine rich repeat protein (SlrP) which inhibits interleukin-1β (IL-1β). This effect seems to maintain the conditions for increasing the opportunity for *Salmonella* to infect other hosts (20). Although these experiments were carried out in preclinical models, the results suggest that glycemia is so important that hosts and infectious agents have developed adaptive strategies to control glucose levels during the progression of an infectious process (11). Moreover, the control of glucose levels may determine the infection course and/or the recovery times (Figure 1); therefore, the understanding of the mechanisms involved on the hijacking of glucose control during infections may have an enormous medical utility.

The relationship between hyperglycemia and susceptibility to infections has been described extensively in diabetic patients. Nevertheless, very few reports have analyzed the appropriate management of glycemia according to the infection type, immunological responsiveness, and clinical variables (i.e., patient age, time or period elapsed with diabetes, etc.). To focus on these aspects, the present review has considered information on: (i) the effect of glycemia on infection outcome and immune cells physiology; (ii) the biochemical alterations in cell physiology during diabetes and/or hyperglycemia; and (iii) the impact of pharmaceutical care interventions for glycemia control on some of the most frequent emerging infectious diseases.

## Methods and Inclusion Criteria

To consider the relevant literature in this theoretical review, we searched for studies published in various databases such as Science direct, Pubmed central and Google Scholar. These databases included any combination of the main key terms “bacterial infections,” “COVID-19,” “diabetes,” “influenza A virus,” “hepatitis B and C viruses,” “human immunodeficiency virus,” and “hyperglycemia” among themselves and with important topics such as: “rheological properties of blood,” “biochemical alterations of diabetic immune cells,” “immune response on hyperglycemic environment,” “hypoglycemic drugs,” “bacterial infection outcome,” and “comorbidities with COVID-19.” Around 600 articles published from 1966 up to 2020 were perused, and only 260 of those articles with experimental and/or theoretical information which related hyperglycemia and/or diabetes to bacterial infections and/or COVID-19 and some other viruses were included in this review.

### The Association Between Hyperglycemia and Common Infectious (Mainly Bacterial) Diseases in Diabetes

#### Effect of Hyperglycemia on the Immune Response

In general, alterations in the immune system during hyperglycemia seem to be associated with mechanisms that include lower secretion of inflammatory cytokines, depression in neutrophils and T cells function, as well as decreases in humoral immunity (21, 22). Moreover, it is documented that hyperglycemia may delay the recuperation of tissues (e.g., via changes in the secretion of growth factors and collagenase levels) (12, 23); this, in turn, may lead to increased susceptibility of these tissues to develop secondary emerging infections (mainly bacterial). Other alterations in the immune response induced by hyperglycemia can be explained by biochemical and/or cellular events, such as: (i) creation of advanced glycation end-products (AGEs), which reduce the expression of myeloid cells surface proteins known as class I major histocompatibility complex (24); (ii) decreased migration of polymorphonuclear leukocytes, chemotaxis, and/or phagocytic activity (25); (iii) inhibition on G6PD (see below) (26); and (iv) increased apoptosis of polymorphonuclear leukocytes and reduced transmigration through the endothelium (27). Clearly, the control of glycemia may be mandatory for dealing with emerging infections (mainly bacterial), in view that: (i) some bacteria grow better in a high glucose environment (28); and (ii) a hyperglycemic state seems to negatively affect the body's ability to respond to antimicrobial therapy (29).

Common infections related to T1DM and T2DM are those of the respiratory and urinary tracts. Indeed, it has been reported that patients with T2DM have alterations in chemotaxis, phagocytosis, antigen presentation and proliferation/function of T cells in response to *Mycobacterium tuberculosis*, which facilitates infection and its symptomatic progression (30). Certainly, the impaired chemotaxis of leukocytes does not depend on the type of diabetes mellitus (31). Other tissues/organs that are also commonly compromised in diabetic patients include the skin, bone marrow, gastrointestinal tract and liver, among others (21, 22, 32). This susceptibility for developing infections may lead to complications in the management of diabetic patients, such as post-operative infections, sepsis, chronic periodontitis, emphysematous cholecystitis, emphysematous pyelonephritis, malignant external otitis, rhinocerebral mucormycosis, gangrenous cholecystitis, and others (21, 22, 32, 33).

It is noteworthy that foot infections are highly common in patients with diabetes, which usually start after a foot wound that eventually leads to ulceration. In this respect, neuropathy seems to be an important component of foot ulceration which, in turn, increases the risk of amputation (34). The wound is predisposed to a loss of sensitivity because of the damage in neuron fibers by pathophysiological mechanisms not fully understood (34–36). It has been suggested that a vascular endothelium damage produced by inflammation and oxidative stress (36) may produce alterations in the microcirculation and, finally, nerve damage (37).

In many cases, these infections cause ischemia at the wound site, ultimately leading to amputations (38). Moreover: (i) immunological disturbances in neutrophil functions such as chemotaxis, phagocytosis and intracellular killing may contribute to exacerbate infections (12, 31, 38–41); and (ii) AGEs may influence the appearance of a chronic immune imbalance by activating pro-inflammatory cells which, in turn, would lead to a chronic subclinical inflammation that hinders the correct function of the immune system to fight infections and to deal with wound healing (42).

Thus, the typical hyperglycemia present in patients with diabetes could be related to an increased risk of different types of infections. Interestingly, cancer patients treated with glucocorticoids showed increased infection rates (43). Indeed, glucocorticoids are direct immunosuppressors that may increase hyperglycemia by hepatic gluconeogenesis and inhibition of glucose intake (43, 44). All these lines of evidence, strongly suggest that hyperglycemia may induce an adequate environment for several infectious pathogens; and hence, a suitable glycemic control would decrease the rate of infection risk (45–49).

Finally, it is logical to assume that the changes in the immune system produced by hyperglycemia as an occasional (transient) event (e.g., stress) should be quite different from the changes induced by a chronic hyperglycemia. Nevertheless, in any hyperglycemic condition (i.e., transient or chronic), patients may be susceptible to some of the same clinical complications, including poor wound healing and an increased rate of infection (50). In fact, acute glucose elevation in critically injured trauma patients may be predictive of infections (51); whereas hyperglycemia at admission (with no indication about the cause) is a predictor of infections in critically ill trauma patients (52). Clearly, chronic hyperglycemia involves compensatory mechanisms (not discussed here) that are absent when it is due to an occasional event; in both cases, normalization of the glucose levels seems to be a useful practice to improve the nosocomial outcome (50, 53, 54).

#### Stress-Induced Hyperglycemia and Infections

Besides diabetes, another condition that commonly predisposes to hyperglycemia is stress. The stress-induced hyperglycemia (SIH) generally refers to a metabolic condition with a transient hyperglycemia associated with clinical illness (55). The SIH is a common problem in patients admitted to intensive care units (ICU) (50), even in the absence of pre-existing diabetes (55), and it is defined as an increase above 200 mg/dL of blood glucose (52, 56, 57).

The SIH is especially dangerous in chronic critical illnesses (58), as the organs' functions become aberrant increasing the risk of death. In less severe cases, SIH seems to affect the normal immune response, since hyperglycemia (as discussed above) is associated with an increased risk to infections (50). Hyperglycemia is certainly related to a higher risk of infections and sepsis in patients of ICU (59), an increased risk of complications in patients who underwent orthopedic trauma surgery (60), and surgical site infections in non-diabetic orthopedic trauma patients (61) (see Table 1).

**Table 1:** Main complications during bacterial infections in diabetic patients.

**Pathogen**	**Emerging disease**	**Main complications in diabetics**	**References**
**RESPIRATORY INFECTIONS**			
*Streptococcus pneumoniae*	Pneumonia	Respiratory failure, pleural effusion, bacteremia, septic shock	(62–64)
*Mycobacterium tuberculosis*	Tuberculosis (TB)	Impaired cell-mediated immunity, renal failure, micronutrient deficiency and pulmonary microangiopathy	(65, 66)
**URINARY TRACT INFECTIONS**			
*Escherichia coli* and *Proteus* sp.	Pyelonephritis	Pherinephric and/or renal abscesses, emphysematous pyelonephritis, renal papillary necrosis, urosepsis, and hemolytic-uremic syndrome	(5, 29, 67–70)
**GASTROINTESTINAL INFECTIONS**			
*Helicobacter pylori*	Gastritis	Macroangiopathy, neuropathy, proteinuria and microalbuminuria	(71–74)
**SKIN AND SOFT TISSUE INFECTIONS**			
*Staphylococcus aureus* and *Staphylococcus epidermidis*	Foot infection	Amputation, osteomyelitis, and death	(13, 75–78)
Combination of one anaerobic and many aerobic microorganisms	Necrotizing fasciitis	Fulminant local tissue destruction, microvascular thrombosis, and systemic signs of toxicity	(79–81)
*Escherichia coli, Klebsiella* sp., *Proteus* sp., and *Peptostreptococcus*	Fournier gangrene	Sepsis, multiple organ failure, and death	(82, 83)
**HEAD AND NECK INFECTIONS**			
*Pseudomonas aeruginosa*	Invasive external otitis	Periostitis, osteitis, chondritis, osteomyelitis, multiple cranial nerve palsies, and facial paralysis	(84, 85)
*Listeria monocytogenes*	Listeriosis	Sepsis, meningitis, hydrocephalus	(86–88)

***References:** (5, 13, 29, 62–80, 82–85, 89–91)*.

A stress response is associated with an increased pro-inflammatory response characterized by the release of several cytokines, including tumor necrosis factor-alpha (TNF-α), IL-1 and IL-6, which are related to insulin resistance (59). Indeed: (i) TNF-α inhibits tyrosine kinases and decreases tyrosine phosphorylation of the insulin receptor (92); (ii) IL-1 suppresses glucose transporter-4 (GLUT-4) translocation by a decreased activation of the phosphoinositide-3-kinase (PI3K) mechanism (93); and (iii) IL-6 increases the release of adrenocorticotrophic hormone (94), with all the scenarios resulting in insulin resistance.

**Figure 2:**
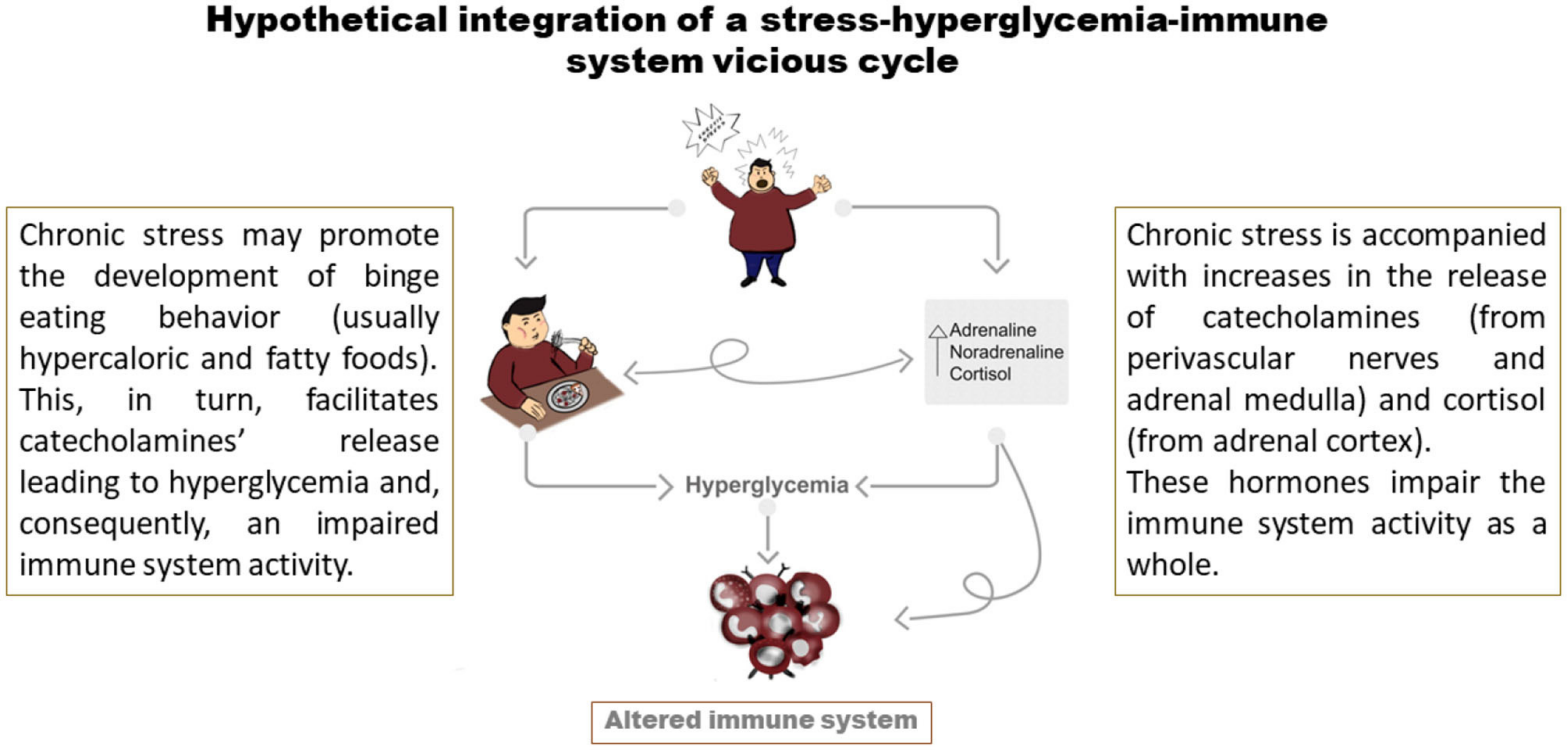
Development of a vicious cycle induced by a stressful lifestyle. Stressors promote the behavior of hypercaloric food binge which, in turn, increases the release of catecholamines and cortisol (immunosuppressants). Subsequently, catecholamines induce glycolysis, reinforce the maladaptive eating patterns, and negatively modulate immune cell activity. On the other hand, cortisol induces chronic immunosuppression.

Additionally, there may be an integration of a vicious cycle when taking a hypercaloric fatty diet, which can also induce an increase in catecholamines' release (95), in combination with lifestyle factors leading to chronic stress (resulting in a plasma increase in catecholamines and cortisol). In this scenario, catecholamines via β-adrenoceptors expressed in adipocytes, liver, skeletal and smooth muscle cells may increase the metabolism of glycogen and triglycerides for increasing blood glucose, fatty acids, glycerol, and other local vascular actions (96–98). Accordingly, this SIH may lead to immunosuppression (see Figure 2).

The contribution of SIH in the diabetic patient is a complex issue that seems to worsen the glycemic status of patients with type 2 diabetes; whereas the autonomic damage induced by neuropathy in type 1 diabetes leads to contradictory and non-conclusive data (99). Patients with necrotizing fasciitis (and diabetes) developed adverse outcomes when SIH generated glycemic gaps with increases in glucose >146 mg/dL (86). Thus, synergy of diabetes and SIH is a phenomenon of high medical impact (both in infectious and non-infectious clinical admission) that remains to be completely characterized (53, 100–104).

### Infectious Diseases in Patients With Diabetes: Exacerbation and Susceptibility to *SARS-CoV-2* and Other Viral Diseases

Diabetes is a condition that may potentiate infectious diseases and predispose patients to acquiring more severe diseases. To support this notion, a recent matched cohort study analyzed the incidence infection rate from 306,011 patients (102,493 patients with type 1 and 2 diabetes), and reported that patients with diabetes (especially type 1) are more susceptible to developing severe infectious diseases (105). In addition, patients with diabetes are more vulnerable to fungal, viral, and bacterial infections than the non-diabetic population, exhibiting a worse prognosis once the infection is installed (106).

According to epidemiological studies, the most common infectious diseases of hospitalization in children, adolescents and adults with diabetes are lower tract respiratory infections (pneumonia, among others), diabetic foot infection, skin and soft tissue abscesses, and urinary tract infections (107, 108). The respiratory tract infections are the primary comorbidity associated with severe or lethal infections that increases hospitalizations in individuals with diabetes (109, 110).

Pneumonia is the hospitalization leading cause of severe lower respiratory tract infections, and it is an important risk factor for infectious illnesses in diabetic patients (111). The main fungal and bacterial pathogens associated with pneumonia infections are Mycoplasma pneumoniae, *Chlamydophila pneumoniae, Legionella pneumophila*, methicillin-resistant *Staphylococcus aureus, Haemophilus influenzae*, and *Streptococcus pneumoniae* (109). This diversity of pneumonia development pathogens may denote a complex biological interaction between wild microorganisms residing in the human body, the host immunophysiology, and the pathogenic pneumonia specificity.

On the other hand, hormones like cortisol, glucagon and catecholamines released during certain conditions, such as trauma, infection and surgery (57) increase gluconeogenesis and decrease peripheral glucose uptake (52). Interestingly, the association between sympathetic hyperactivity (e.g., induced by chronic stress), hyperglycemia, hypothermia and immunosuppression of the acquired immunity seems to be mainly mediated by activation of α-adrenoceptors (112, 113).

Emerging global health studies have reported that other respiratory tract infections with high mortality rate in patients with diabetes, besides pneumonia (114), are those promoted by viral agents. These include the influenza viruses, the Middle East respiratory syndrome coronavirus (MERS-CoV), the severe acute respiratory syndrome coronavirus (SARS-CoV) and, most recently, the SARS-CoV-2 (see below), the last viral infection outbreak across the globe (7, 17, 115, 116).

#### SARS-CoV-2 and Other Viral Diseases: Impact of Hyperglycemia

Patients with hyperglycemia have been reported to be susceptible to develop a severe form of COVID-19, which is a risk factor for fatality (6, 17). Newsworthy, diabetes provides a ~3-fold higher risk of fatality as compared to the non-diabetic population among the COVID-19 sufferers (7). Moreover, diabetes increased the length of hospital stays for COVID-19 patients from 9.8 days in non-diabetic patients to 14.4 days in diabetic patients in a retrospective cross-sectional study that was conducted in England (14). In this regard, it has been described that a proper control of glycemia by antidiabetic drugs can be beneficial in reducing the risk of death in diabetic patients with COVID-19 (16). Indeed, it was inferred that DPP-4 inhibitors might be beneficial to prevent or treat COVID-19 disease (117). Although this certainly opens a new field of interest in the treatment of SARS-CoV-2 pneumonia, further studies and research are required on this topic.

Bacterial infections are frequently identified after typical viral respiratory infections and they are important causes of morbidity and mortality. In patients with COVID-19, bacterial comorbidity has been reported to be low (i.e., an overall proportion of 6.9 %) in a recent metanalysis reported by Langford et al. (118). Notoriously, the comorbidity was slightly higher in critical patients (i.e., 8.1%) (118).

DM, hypertension, cardiovascular diseases, and obesity are the top four comorbidities worldwide associated to critically ill patients with COVID-19 and mortality (15, 119–121). Indeed, 5 to 10% of patients with SARS-CoV-2 pneumonia require intensive care unit (ICU) admission and mechanical ventilation. Patients requiring invasive mechanical ventilation are strongly related to poor outcome with high mortality rate in Chinese and American populations (122, 123).

Unfortunately, studies on the unfavorable outcomes and mortality rate related to pathogenic co-infections that worsen respiratory tract function in people with diabetes and COVID-19 infection are limited (124). However, bacterial, and viral pathogenic co-infections have been studied in patients with SARS-CoV-2 pneumonia requiring ICU admission. These studies showed that methicillin-sensitive *Staphylococcus aureus, Haemophilus influenzae, Streptococcus pneumonia*e, *Enterobacteriaceae, Pseudomonas aeruginosa, Moraxella catarrhalis*, and *Acinetobacter baumannii* were the 28% of bacterial strains isolated by experimental laboratory procedures (cultures or PCRs assays) (125). Importantly, no viral co-infection has been detected in the critically ill COVID-19 patients, supporting the idea that respiratory infections are often depending on combinatorial factors associated to geography, season, human physiology, and behavior, as well as pathogenic interactions. Therefore, it is mandatory to determine the different biological mechanisms used for each viral infection and/or co-infection of pathogens that aggravate states of health or disease to pursue appropriate treatments.

Admittedly, pathogenesis of COVID-19 viremia remains unclear. However, some lines of evidence suggest that high levels of systemic glucose increase glucose concentration in the epithelial secretion of the respiratory tract, disrupting the orchestration of the innate and humoral immunological response. This includes, in particular, hyperglycemia-induced changes in coagulation, worsening of endothelial function, and reproduction of inflammatory cytokines (126).

Data about hospitalization for infectious diseases in diabetic and non-diabetic subjects have been associated with various hyperglycemic conditions on admission, increasing poor outcomes and mortality rates. Moreover, hyperglycemia on admission was clearly associated with undiagnosed DM, strongly suggesting that an optimal glycemic control that reduces glycemic fluctuations during hospitalization should be a beneficial clinical practice for viremia control (127).

The American Diabetes Association (A.D.A.) recommends a blood glucose level of 140 to 180 mg /dL (7.8 to 10.0 mmol/L) for most critically ill patients and patients who are not in good health. Glycemic control during clinical procedures could be accompanied by insulin therapy if the hyperglycemia persists starting at a threshold ≥ 180 mg/dL (128). Thus, patients with COVID-19 and with/without DM should have a well-controlled blood glucose (129).

#### Influenza A Virus

The influenza A virus (IAV) induces a self-limited infection in most patients, which is characterized by several symptoms such as myalgia, fever, and dry cough (130). Nevertheless, patients with diabetes experience a more severe type of this disease (131) that is represented by a triple risk of hospitalization and double risk of fatality compared with non-diabetic sufferers (132). Despite the fact that IAV infects upon 15% of the world's population every year (133), the full mechanisms underlying its pathogenesis, especially on patients with diabetes, remain thus far inconclusive. In this sense, Hulme et al. (132) reported that the IAV infection in hyperglycemic conditions increases the endothelial damage leading to a pronounced inflammatory response; this explains, at least in part, the severity of the symptoms in patients with diabetes (132). In support of this notion, Kohio and Adamson (134) reported an enhanced IAV replication rate in pulmonary epithelial cells under elevated glucose concentrations *in vitro*.

Admittedly, the specific mechanisms that underline susceptibility factors for viral infections development in patients with diabetes (Table 2) are poorly understood. However, an experimental study (132) that used an *in vitro* and *in vivo* model of pulmonary epithelial-endothelial cells exposed to a high glucose concentration (12 mM) demonstrated an increased barrier damage after co-cultured cells were infected with IAV; this, in turn, augmented pulmonary edema associated with a pro-inflammatory response (132). Thus, controlling hyperglycemia seems to be important for hospitalized patients with severe viral infections and diabetes (144).

**Table 2:** Main complications during viral infections in patients with diabetes.

**Pathogen**	**Emerging disease**	**Main complications in patients with diabetes**	**References**
**RESPIRATORY INFECTIONS**			
Influenza virus	Pneumonia	Risk of admission to the intensive care unit, fatal outcome after infection, increasing influenza severity, and secondary bacterial infections	(130, 135, 136)
Severe Acute Respiratory Syndrome Coronavirus 2 (SARS CoV2) virus	COVID-19	Inflammatory storm in atherosclerotic plaques, increased viral secondary infection to lung, acute respiratory distress syndrome, acute renal failure, acute cardiac injury and heat failure, and increased risk for patient mortality	(137, 138)
**LIVER INFECTIONS**			
Hepatitis B virus^a^	Hepatitis B	Elevated serum triglyceride level, blood glucose abnormalities steatosis and cirrhosis	(22, 139)
Hepatitis C virus	Hepatitis C	Reduced rate of sustained virological response, progression to fibrosis and cirrhosis, and higher risk for development of hepatocellular carcinoma	(22, 140, 141)
**OTHER INFECTIONS**			
Human immunodeficiency virus	HIV/AIDS	Hypertension, dyslipidemia, and acute myocardial infarction	(142, 143)

a *The studies about the relationship between Hepatitis B virus and T2DM are not consistent*.

***References:** (22, 130, 135–143)*.

#### Hepatitis B and C Viruses

The Hepatitis C virus (HCV) and Hepatitis B virus (HBV) are known causes of hepatic decompensation, liver cirrhosis, and hepatocellular carcinoma (HCC), being two major public health problems worldwide (145–147). The evidence for a link between HCV and DM has been proposed several decades ago (148). In this sense, based on the meta-analysis of Gou and colleagues (145), patients with T2DM are more prone to HCV infection (~3.5-fold increase) compared with the risk in the non-diabetic group. In the case of HBV, the diabetic condition predisposes to acquiring the infection (147, 149). Moreover, there is a high association between diabetes and the higher risk for a worse outcome of HCV and HBV infection (139, 141, 150, 151). HCV patients with diabetes have a higher incidence of HCC compared to non-diabetic HCV patients (radio 1.73) (152). Interestingly, the use of several hypoglycemic drugs improves the prognosis for this type of cancer (153, 154).

#### Human Immunodeficiency Virus

Human immunodeficiency virus (HIV) increases the risk for developing T2DM (155, 156). Likewise, patients with HIV are prone to diabetes in younger people and in the absence of obesity (157, 158). In this sense, several hypotheses have been proposed to understand the mechanisms for this link, including the effects of antiretroviral drugs (ARVD), lipodystrophy, co-infections, and autoimmunity (156). The use of ARVD in patients with HIV, which include atazanavir, darunavir, and saquinavir, interfere with the GLUT-4 dynamics by increasing insulin resistance and reducing insulin secretion (159). On the other hand, it has been recognized that the HIV infection and/or its treatment can induce lipodystrophy (i.e., an abnormal distribution of fat in the body); this raises the levels of TNF-α which, in turn, contributes to increasing insulin resistance and finally triggering diabetes (160). The third hypothesis to understand the relation HIV-diabetes includes the co-participation of HCV; in this sense, the increased intrahepatic TNF-α may be a trigger to develop diabetes (161). Finally, the autoimmune hypothesis explains that some HIV-patients may undergo beta cell destruction, developing the autoimmune diabetes observed in some HIV-infected patients (162).

### Physicochemical Changes During Hyperglycemia: Effects on the Immune System

#### Rheological Properties and Blood Viscosity

Rheological properties of blood may impact function, metabolism, motility and even the latency for clearing toxins of blood cells (163, 164). Changes in rheological conditions have been reported during diabetes and hyperglycemia, which may alter red blood cells physiology and the local microcirculation (163, 165). Indeed, some of the blood rheological properties that have been reported to be disturbed during hyperglycemia and/or diabetes include: (i) an increment in serum osmolarity (166); (ii) erythrocyte deformation that is produced by glycosylation of membrane proteins (167, 168); (iii) changes in *p*H (169); and (iv) an increase in blood viscosity (164, 165). All these alterations may impair the immune system activity and could explain the impact that glycemia has on the clinical outcome (Figure 1).

Furthermore, increased blood viscosity may lead to hemoconcentration and vasodilatation that increases edema (164). In close connection with this response, coagulation directly affects blood viscosity, increasing the risk for developing microangiopathy (168). In fact, anomalous erythrocyte deformability and platelet aggregation impair microcirculation, which leads to hypoxia in hyperglycemia and diabetes (170, 171). In this sense, a decrease in oxygen supply could impair the immune response because in those cells oxygen is essential for destroying infectious microorganisms (172). As a result, oxygen supplementation: (i) avoids surgical infections during the perioperative period (173, 174); and (ii) can be used to prevent infections and promote wound healing (175).

To round off and complete the above rheological scenario, it is to be noted that the concentration of fibrinogen and globulins are also important factors involved in blood viscosity (168). In fact, an increase in plasma fibrinogen in diabetic patients is a determining factor for blood viscosity (176). This, in turn, will alter oxygen supply resulting in an impaired immune response.

#### *p*H

Any change in *p*H may be detrimental for the proper functioning of the whole body (169), including the diabetic sufferers. In this sense, diabetic ketoacidosis (DKA) is a common hyperglycemic condition that affects both T1DM and T2DM patients, resulting in a decreased venous blood *p*H (below 7.3) (177). DKA results from an altered metabolism of glucose mainly produced by a decreased or abolished production of insulin (178). This, in turn, promotes the metabolism of triglycerides into glycerol and fatty acids, with the latter being further oxidized to ketone bodies, mainly acetoacetate and β-hydroxybutyrate (178, 179). Ketone bodies are weak acids that weigh down blood buffering capacity (carried out by bicarbonate anion), altering *p*H and resulting in a metabolic acidosis (177, 180).

As the most severe complication of DM, patients with DKA have more difficulty to handle infections (179, 181). Admittedly, it is not clear whether DM may increase the susceptibility for all infections; however, many of them (mainly the bacterial ones) are more severe, frequent and/or typical of diabetic patients (21). For instance, some of the most common infections in these patients are pneumonia and urinary tract infections (179, 182), as well as other infections difficult to manage, such as mucormycosis (183, 184), aspergillosis (185), tuberculous meningitis (186), and pulmonary coccidioidomycosis (187).

Several reports have shown the role of *p*H in the immune response. For example, with a *p*H below 6.5: (i) the mobility of polymorphonuclear leukocytes was impaired (188), which could result in delayed migration of leukocytes; (ii) chemotaxis was inhibited (188, 189); and (iii) the production of superoxide anion was decreased in neutrophils (190), resulting in an impaired “respiratory burst” (191). However, phagocytosis in bovine neutrophils was hardly affected when they were challenged with *Staphylococcus aureus* at acidic *p*H (192). Moreover, Loeffler et al. (193) reported an inhibition in lymphocytes proliferation induced by interleukin-2 (IL-2) at acidic *p*H. Nevertheless, only some functions seem to be affected in lymphocytes at an acidic *p*H, namely, at *p*H 6.7 (as compared with *p*H 7.1) an increase in lymphocytes mobilization was reported (194, 195). A possible explanation for this finding is that every cell type and specific functions are differentially altered by *p*H gradients. Obviously, further studies are required to understand the molecular mechanisms underlying each cellular type and the corresponding physiological phenomena.

Other important alterations induced by hyperglycemia in the circulatory system are related to a miss-functionality of the enzymatic machinery of blood cells, including Na^+^/K^+^-ATPase activity and glucose-6-phosphate dehydrogenase (G6PD) (see below).

#### Alterations in Na^+^/K^+^-ATPase Activity

Na^+^/K^+^-ATPase is a transmembrane protein responsible for maintaining intracellular Na^+^/K^+^ balance by generating the gradients of Na^+^ and K^+^ (196). This enzyme is expressed ubiquitously in almost all cell types, regulating a plethora of functions such as the reabsorption of glucose and amino acids (which depends on a Na^+^ gradient) in distal convoluted tubule, motility in sperm cells, action potentials in synaptic neurons, etc. (197). In erythrocytes, this enzyme is involved in maintaining their volume and water homeostasis (198); while in lymphocytes, their proliferation induced by a variety of stimulus is dependent on Na^+^/K^+^-ATPase activity (199). Interestingly, Na^+^/K^+^-ATPase activity is decreased in the erythrocytes from T2DM sufferers (198, 200), but its expression remains unaltered (201). These findings suggest that the activity of Na^+^/K^+^-ATPase may be used as a potential biomarker for detecting early phases of T2DM (202). Within this context, one theory that explains the effects of hyperglycemia on Na^+^/K^+^-ATPase is by glycosylation, which induces the impairment of the ATPase activity in erythrocytes (202). In fact, this enzyme has several glycosylation sites located at β-subunits, some of them related to protein maturation (203) and other functional processes (197). These lines of evidence show the importance of glycosylation in Na^+^/K^+^-ATPase activity.

On the other hand, Na^+^/K^+^-ATPase partake in the functionality of immune cells (199, 204, 205). Indeed, proliferation of lymphocytes is dependent on Na^+^/K^+^-ATPase activity (199) and the expression of nuclear factor of activated T cells transcription complex (NFAT) of thymocytes (206); this factor is essential for the production of Interleukin-2 (207), a cytokine produced by lymphocytes during a microbial infection (208). Hence, immunologic and hematologic deficiencies in diabetic patients are related to multiple alterations, which may include aberrant activity of the Na^+^/K^+^-ATPase.

In agreement with the above findings, a reasonable possibility to explain the alterations in immune system activity during diabetes is that the Na^+^/K^+^-ATPase activity could be equally decreased in both lymphocytes and erythrocytes (since these cell types are in the same environment) (201). Besides this, protein glycosylation can occur by enzymatic, but also by non-enzymatic ways; in this respect, glucose is chemically attached to proteins by Schiff base and Amadori product adducts, resulting in a variety of biological effects, including inactivation of enzymes (209), such as Na^+^/K^+^-ATPase. It has even been reported that a deficiency in glucose-6-phosphate dehydrogenase, an enzyme altered in diabetes, increases protein glycosylation (210), supporting the idea previously proposed (see below).

#### Glucose-6-Phosphate Dehydrogenase

Glucose-6-phosphate dehydrogenase (G6PD) is an enzyme expressed ubiquitously in all mammalian tissues. It plays an important role in the pentose pathway catalyzing the first reaction in this metabolic route, which is necessary to convert glucose into pentose sugars (211). This pathway produces nicotinamide adenine dinucleotide phosphate hydrogen (NADPH), an antioxidant molecule that catalyzes the reaction to regenerate reduced glutathione (212).

Many studies have reported the importance of G6PD in antioxidant defense against toxicity of reactive oxygen species (ROS) (211, 213). Interestingly, a relationship is established between diabetes and a decrease in G6PD activity in a variety of cells from rats (212) and humans (214). Additionally, this enzyme plays an important role against infections (213, 215) and in T cell proliferation (216). In keeping with this view, a deficiency of this enzyme in leukocytes is related to serious infectious diseases, such as chronic granulomatous disease (172, 217).

Admittedly, the specific molecular mechanisms that explain the effects of chronic hyperglycemia on G6PD activity in immune cells remain uncertain. For example, Xu et al. (26) showed evidence of inhibition of this enzyme via phosphorylation by protein kinase A in kidney cortex of diabetic rats pretreated with streptozotocin. Similar results were observed in aortic endothelial cells cultured under hyperglycemic conditions (218). Another possibility to explain the effect of glucose on G6PD activity is via protein glycosylation produced by a high glucose concentration (219).

In summary, the above physicochemical alterations resulting from hyperglycemia impair the immune response, predisposing diabetic subjects to acquire infections as well as exacerbates them.

### Potential Benefits of Hypoglycemic Drugs on the Outcome of Clinical Infections

#### Hypoglycemic Drugs and Their Clinical Effects on Bacterial Infections

An uncontrolled blood glucose level is associated with an increase in microvascular and macrovascular complications in diabetic patients (220). Likewise, a hyperglycemic state results in multiple consequences, including osmotic diuresis, fluid/electrolyte imbalance, poor wound healing, impaired immune response, and increased susceptibility to infections, among others (22, 221). Accordingly, these pathophysiological conditions have led to the implementation of therapeutic strategies for a tight glycemic control in patients with T2DM, resulting in the development of the so-called glucose-lowering drugs (i.e., Oral Antidiabetic Drugs; OADs).

Several lines of evidence have shown that the use of OADs to maintain tight blood glucose concentrations between 80 and 110 mg/dl decreases infection-related complications and mortality (see Table 3). For example, metformin, which is the first-line pharmacological agent for T2DM treatment (233), reduced airway glucose permeability and prevented the higher load of *Staphylococcus aureus* (*S. aureus*) induced by hyperglycemia (224). Similarly, metformin pre-treatment inhibited the glucose-induced growth of *Pseudomonas aeruginosa*, increased transepithelial electrical resistance (TEER) and decreased glucose flux in an epithelial cell culture model (234). In this sense, mutants of genes affecting glucose uptake of *P. aeruginosa* decreased the bacterial loads on streptozotocin-induced hyperglycemic mice compared to control.

**Table 3:** Pharmacodynamics of some hypoglycemic drugs and their reported effects on infectious processes.

**Drug**	**Mechanism of action**	**Reported effects on infectious processes**
Metformin	It decreases hepatic production and intestinal absorption of glucose with an improvement in insulin sensitivity (222)	Decreased risk of chronic lower respiratory diseases (223) Reduced infection with *S. aureus* and *P. aeruginosa* in mice (224) Inhibited growth of *P. aeruginosa* in airway epithelial cell line (Calu-3) *in vitro* (225) Reduced risk of *Mycobacterium tuberculosis* infection compared to those which received sulfonylureas as initial treatment for diabetes (226) Reduction of ~20% in the risk of sepsis (227)
Sulfonylureas	It increases insulin secretion via ATP-sensitive potassium channel- pathway (228)	Inefficient to reduce the risk of sepsis (227)
Acarbose	It inhibits the alpha-glucosidase enzymes in the small intestine, delaying the breaking down complex carbohydrates and sucrose (229)	N/D
Thiazolidinediones	Interaction with the nuclear peroxisome proliferator-activated receptor-gamma (PPAR- γ), regulating the transcription of several insulin responsive genes (230)	Moderate reduction in the risk of sepsis (227)
Dipeptidyl peptidase 4(DPP-4) inhibitors	It increases insulin secretion and inhibits the release of glucagon (231)	No association between DPP-4 inhibitors and risk of sepsis (227)
Sodium-glucose co-transporter 2 (SGLT2) inhibitors	Inhibit renal reabsorption of glucose (232)	N/D

***References**: (222–232)*.

*N/D, not determined*.

Interestingly: (i) metformin pre-treatment of hyperglycemic animals reduced both airway glucose and bacterial load (234); (ii) the incidence of tuberculosis has been related to abnormal glucose levels, whereas metformin is a protective agent in the treatment of tuberculosis in diabetic patients (235); (iii) metformin treatment was also associated with an increased risk of bacterial pneumonia in patients with chronic obstructive pulmonary disease from a nationwide cohort study (Taiwan) (236); and (iv) pneumonia is a swelling disease usually caused by a bacterial infection commonly associated with diabetic patients (237).

Consistent with the above findings, diabetic patients with community-acquired pneumonia (CAP) developed worse results and longer hospital stays in comparison to patients with CAP without diabetes (238); accordingly, it is important to discuss the relationship between the use of OADs and pneumonia. Indeed, these data support airway glucose as a critical determinant of increased bacterial load during diabetes (225).

Moreover, Mendy et al. (223) analyzed data from the National Health and Nutrition Examination Survey during 1988–1994 and 1999–2010 for participants aged 40 years or older who had diabetes and were followed up for mortality through 2011. Their results showed that metformin was associated with a decreased risk for chronic lower respiratory diseases (CLRD) mortality in the overall population (HR: 0.39, 95% CI: 0.15–0.99) and among participants with baseline CLRD (HR: 0.30, 95% CI: 0.10–0.93) (223).

Likewise, Pan et al. (226) investigated the effect of metformin vs. sulfonylureas on tuberculosis risk in patients with T2DM. The study demonstrated that patients with T2DM treated with metformin in the initial 2 years, had a significant reduced risk of tuberculosis as compared to those receiving sulfonylureas as initial treatment (226).

Furthermore, Shih et al. (227) reported the relationship between the use of AODs and the risk of hospitalization for sepsis. The authors found that the use of metformin was associated with ~20% reduced risk of sepsis as compared with non-use. In contrast, meglitinides and sulfonylureas were associated with increased risk of future sepsis events, but this association was not evident among recent and current sulfonylurea users. Moreover, the DPP-4 inhibitors and thiazolidinediones on sepsis were neutral, nevertheless, the occurrence of sepsis in current thiazolidinediones users was reduced (227).

On the other hand, some studies have shown that pretreatment with dapagliflozin, a sodium-glucose co-transporter 2 inhibitor, reduced blood and bronchoalveolar lavage glucose concentrations and *P. aeruginosa* CFU in leptin receptor-deficient (db/db) mice, as compared to those seen in wild type (WT) mice (239).

In summary, the available evidence thus far has established the increased susceptibility to certain types of infections related to hyperglycemia in T2DM. Clearly, further studies on the mechanisms regulating OADs and bacterial action on specific tissues/organs are required. Such studies could yield potential alternatives to prevent/suppress hyperglycemia and bacterial infections.

Moreover, the risk for developing infections is increased in hyperglycemic environments, where there is a lower production of interleukins, a reduced chemotaxis and phagocytic activity, and a gastrointestinal dysmotility (22). The use of specific OADs such as metformin is associated with reduced hospital-treated infections, septicemia prognosis, and some kinds of respiratory illnesses (224, 240). Indeed, another study in diabetic patients has shown a reduction in autoimmune diseases by an acute intervention with OADs, such as DPP-4 in combination with other hypoglycemic drugs (241). One mechanism that may improve those immune response effects is through GLP-1 action that induces insulin secretion and inhibits glucagon secretion, ameliorating the glycemic variability (242).

To conclude this section, it is to be noted that experimental anorexia seems to play an important protective role in supporting the recuperation of bacterial infections (19). Moreover, mortality in critical illnesses (e.g., sepsis, severe burning, etc.) may increase via alterations in immune cell activity that, in turn, may be mediated by the release of stress hormones (cortisol, catecholamines, etc.) and the hyperglycemia that these hormones induce (57). As hyperglycemia impairs periphery glucose usage, administration of insulin improves cellular uptake and attenuates the inflammatory response (57).

A higher risk of CAP was found with other OADs, except with dipeptidyl-peptidase 4 (DPP-4) inhibitors (237). Indeed, in a retrospective cohort and a meta-analysis study, DPP-4 inhibitors failed to increase the risk of pneumonia during diabetes (243). These controversial data about the use OADs and the outcome of bacterial infections in diabetic patients point out the necessity for more detailed analyses and clinical observations.

In view that hyperglycemia may be a determinant factor in the outcome for bacterial infections, any effort for controlling the increases glucose levels is valuable. Another interesting approach may be the supplementation with calcium and vitamin D because it decreases insulin resistance and hyperglycemia (244); nevertheless, some strategies must be considered to ponder the risks and benefits.

#### Further Considerations

Hypoglycemia occurs when there exists a lack of adequate food intake, excessive exercise, a stressful experience, excessive alcohol consumption, concurrent infections, severe digestive and urologic diseases, and/or after taking antidiabetic medications (245). This suggests that hypoglycemia is an endocrine alarming signal that is triggered to level the required concentrations of blood glucose in the body.

Considering that fasting plasma glucose is normally maintained between 70 and 99 mg/dL (2), a biomarker associated with high blood glucose levels is HbA1c, whose normal range is between 4 and 5.7% in healthy people. Less than 7% of HbA1c is found in controlled people with diabetes, and above 8% is found in people with uncontrolled diabetes (246). Low or high levels of HbA1c have been related to severe hypoglycemic episodes with a glucose-lowering regimen in patients with diabetes (247).

Glycemic control and reduction of hyperglycemia or hypoglycemia events are the main challenges in the clinical experience to achieve decreases of blood glucose variability (248). Indeed, levels of glucose and its constant fluctuations are good indicators of organ dysfunctions such as those associated with infections (249). Patients with diabetes often suffer from chronic low-grade infections such as periodontitis and foot ulceration. Surgery-site infections and susceptibility to septic shock increase with pre and post-operative glucose levels and their variability (250, 251), suggesting that glucose monitoring is one of the key elements in hyperglycemia and hypoglycemia management diseases where the immune system is compromised.

Glucose variability is currently considered more deleterious than chronic hyperglycemia in the development of diabetes-related complications (252). However, some studies suggest that an intensive glucose control does not improve some of the diabetes-associated complications such as cardiovascular failures, raising the mortality rate (253). Furthermore, a tight glucose control induces hypoglycemic episodes and the increased response of the immune system, impacting on coagulant factors, pro-inflammatory cytokines, proatherogenic cell adhesion molecules, and nitric oxide-mediated vasodilatation. Innate immunity response is activated nearly after acute or chronic infections are experienced by diabetes sufferers. For this, the study of the suppression of innate immune system is a key factor, since it exacerbates the inflammatory response after an acute hypoglycemia episode, inducing prothrombotic changes and increasing platelet reactivity (254).

Another consideration is that under normal and pathophysiological metabolic functions, individuals course with glucose swings during the day (255), correlating them with the gastric emptying rate and postprandial glucose levels. Glycemic fluctuations are limited by low glucose levels that slow the gastric emptying or by high glucose that accelerate it (256). However, a hypoglycemic state promotes reverse effects; hence, the gastric emptying is accelerated and the absorption speed of nutrients is increased to reach the physiological glycemic levels, suggesting that gastrointestinal motility and gastric motor function are important factors to consider for a therapy of glycemic control (257).

During physiological gastric emptying, carbohydrates and proteins are evacuated faster than lipids for their caloric content. The evacuation of these macronutrients begins at 20 to 40 min after food intake and when they reach the intestine, incretin hormones are secreted to blood. Glucagon-like peptide-1 (GLP-1) is an incretin hormone that stimulates insulin secretion, reduces glucagon secretion, and delays gastric emptying in a glucose-dependent manner (256, 258).

Significantly, glucose-lowering therapies through the use of diverse drug classes have been reported as an important source of heart failure risk, particularly with differential effects on insulin (259). Consequently, older patients are the most affected population, especially if a diminished food intake, excessive alcohol use, combination of non-prescribed medications, concomitant infections, and diabetic complications are also taken into account (260). Because of this, an intervention with a forced hypoglycemia should be considered with caution according to disease timing, age, nutritional behaviors, type of medications and concomitant infections.

## General Conclusion

Hyperglycemia clearly induces physiological and immunological disorders in body tissues/organs that may predispose and exacerbate some infectious diseases. Therefore, the control of glucose levels could be an alternative tool to contribute to the fight against infections not only in diabetic patients, but also in other conditions that induce hyperglycemia, such as SIH. In addition, several studies have shown the potential benefits of controlling it (e.g., pharmacological approaches), opening a new option to improve the outcome of some infections (bacterial and viral). It is worth noting that the authors of this review agree that glycemic control is necessary as part of good intervention strategies to treat current and emerging infectious diseases. Admittedly, the clinical evidence for reducing glycemic exposure requires more supportive data, specifically for hypoglycemia as a tool to fight infections in humans. Notwithstanding, this review summarizes enough preclinical evidence to increase our chances of beating infections by focusing on the key role of glycemic control.

## Author Contributions

JC-R, CE-G, and BM-C developed the central idea of this article and wrote the manuscript. BM-C proposed the central idea, made the graphs, and obtained funding. PV-L, EC-S, AL-B, and AQ-S provided original ideas, developed some sections, and reviewed the manuscript. CMV and JM-R discussed the central ideas, reviewed, edited, and corrected the manuscript. All authors contributed to the article and approved the submitted version.

## Conflict of Interest

The authors declare that the research was conducted in the absence of any commercial or financial relationships that could be construed as a potential conflict of interest. The handling editor and reviewer AV declared a shared affiliation with one of the authors EC-S at time of review.
